# Effect of Topography and Physical Stimulus on hMSC Phenotype Using a 3D In Vitro Model

**DOI:** 10.3390/nano9040522

**Published:** 2019-04-03

**Authors:** Deepak Kumar, Stuart A. Cain, Lucy A. Bosworth

**Affiliations:** 1 School of Materials, Faculty of Science and Engineering, University of Manchester, Manchester M13 9PL, UK; deepak.kumar@dpag.ox.ac.uk; 2 Department of Physiology, Anatomy and Genetics, South Parks Road, University of Oxford, Oxford OX1 3QX, UK; 3 Division of Cell Matrix Biology and Regenerative Medicine, Faculty of Biology, Medicine and Health, University of Manchester, Manchester M13 9PL, UK; Stuart.A.Cain@manchester.ac.uk; 4 Department of Eye and Vision Science, Institute of Ageing and Chronic Disease, University of Liverpool, Liverpool L7 8TX, UK

**Keywords:** electrospinning, hydrogels, composites, human mesenchymal stem cells, extracellular matrix, mechanical stimulation

## Abstract

This communication reports the first comparative study addressing the effects of both structural architecture and mechanical loading on human mesenchymal stem cells (hMSC) positioned at the interface of a 3D in vitro model composed of a nanofibre/hydrogel laminate composite. hMSC phenotype was affected by both stimuli over a seven-day period. Cells were orientated parallel to the underlying fibre direction irrespective of environment (electrospun 2D fibre sheet or laminate 2D sheet with collagen gel layer). Application of cyclical tensile force (5% strain, 1 Hz, 1 h per day) encouraged hMSCs to remain at the fibre/gel interface, whereas cells cultured in static conditions migrated from the interface to the upper hydrogel layer. Depending on the stimulus applied, hMSCs presented an up-regulation in gene expression, indicative of several cell lineages, with those cultured at the interface and physically stimulated expressing markers indicative of angiogenesis, osteogenesis, and tenogenesis. This study highlights the importance of developing biomaterial scaffolds with environmental cues to specifically drive cells towards the tissue intended for bioengineering.

## 1. Introduction

**Figure 1:**
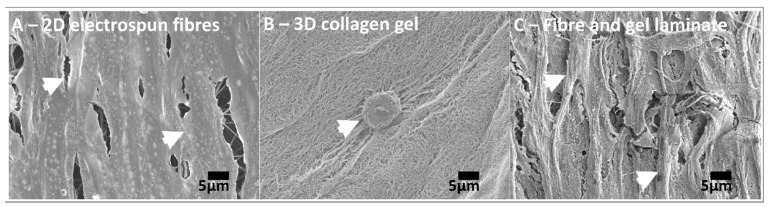
**Scanning Electron Microscope** (SEM) images: (**A**) flattened, elongated fibroblasts on electrospun fibres, (**B**) rounded fibroblast within a collagen hydrogel, (**C**) rounded and elongated fibroblasts on electrospun fibre/hydrogel composite (gel removed for imaging). Scale = 5 μm, arrows indicate position of cells.

Electrospinning and hydrogel technologies are commonly selected in the biomaterial and tissue engineering field because they create scaffolds that closely replicate the extracellular matrix (ECM) [1,2]. Although these technologies have greatly advanced the field, few continue along the translational pipeline towards clinical use. This is largely attributable to inherent disadvantages, whereby cells are unable to invade electrospun nanofibres without compromising structural integrity and hydrogels are either too weak or too rigid [3]. Greater emphasis has been placed on the development of smart nanofibre/hydrogel composites to confer appropriate structural properties and better mimicry of native tissue ECM, which is itself a natural composite of fibres held within a gelatinous matrix. We have previously demonstrated tendon fibroblasts to recover a morphology closer to that observed in vivo when cultured within laminate composite structures when compared to fibres or hydrogel alone (Figure 1). 

Using a novel, 3D, dynamic, in vitro model that better simulates the natural tissue environment, we investigated the behaviour of bone marrow-derived human mesenchymal stem cells (hMSCs), located at the interface of nanofibre-hydrogel laminate composites.

## 2. Materials and Methods

**Figure 2:**
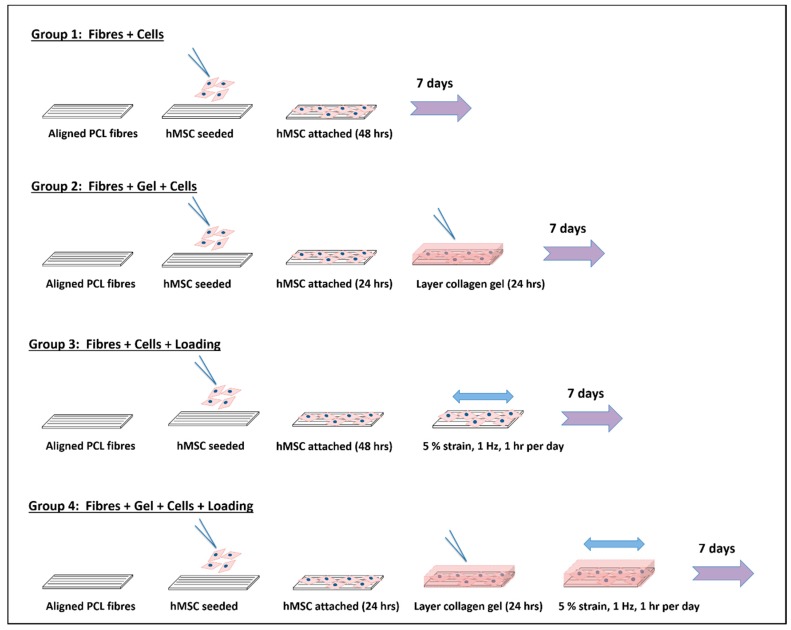
Schematic of experimental set up for four test groups seeded with human mesenchymal stem cells (hMSC).

Nanofibre sheets were prepared by electrospinning a 10% *w*/*v* poly(ɛ-caprolactone) (PCL; Purasorb PC12; Corbion, Netherlands) solution dissolved in 1,1,1,3,3,3-hexafluoroisopropanol (>99% purity; Sigma, UK) targeted towards an earthed rotating mandrel (600 RPM) with the parameters of 20 kV applied voltage, 1 mL/h flow-rate, and 20 cm distance between polymer feed and mandrel. Aligned fibres were removed and cut into 1 × 2.5 cm rectangles (orientation parallel to long-edge). Fibre mats were disinfected in ethanol (VWR, UK) as previously described [4]. hMSCs (P4; PromoCell, Germany) were seeded onto the fibre mats (24 × 10^3^ cells/cm^2^). Four test groups were established (*n* = 3; Figure 2): Group 1 comprising fibres only, static conditions (FS); Group 2 comprising fibres and gel layer, static conditions (FGS); Group 3 comprising fibres only, dynamic loading (FL); Group 4 comprising fibres and gel layer, dynamic loading (FGL). For Groups 2 and 4, a collagen hydrogel layer (300 µL; rat tail, type I collagen), prepared per manufacturer’s instructions (Merck Millipore, UK), was evenly deposited on top of the fibres and cells 24 h post-seeding to create a laminate composite structure. Groups 1 and 2 were held under static conditions within 6-well-plates. Groups 3 and 4 were individually gripped within a Mechanoculture MCT6 loading system (CellScale; Ontario, Canada) and cyclically loaded (5% strain, 1 Hz, 1 h per day). hMSCs had been seeded for 48 h prior to experimental start, which was a subsequent seven-day period. 

After seven days, samples were evaluated by qRT-PCR and confocal microscopy. For qRT-PCR, samples were washed with Phosphate Buffered Saline solution (PBS; Sigma) and stored in Trizol (Sigma) at −80 °C. Sample RNA extraction was performed as previously described [5]. One-step qRT-PCR was performed using QuantiFast SYBR green enzyme (Qiagen, UK) according to manufacturer’s instructions. QuantiTect Primers (forward and reverse primers) included: GAPDH (housekeeping gene; QT00079247), RUNX2 (bone transcription marker; QT00020517), VEGF (Vascularisation transcription marker; QT00013783), ELN (Elastin; QT00034594), SOX9 (Cartilage transcription marker; QT00001498), COL2A1 (Type 2A1 collagen, cartilage transcription marker; QT00049518), and TNC (Tenascin-C, tenogenic transcription marker; QT00024409). Primer annealing temperature was 60 °C for 30 s. Raw C_T_ values were normalised to GAPDH to obtain ΔC_T_ values, converted to ΔΔC_T_ values, and presented as fold-change relative to GAPDH. 

For confocal, samples were fixed in 4% paraformaldehyde (Sigma) (1 h at room temperature (RT)), washed in PBS, and permeabilised using permeabilisation buffer (0.1% Triton X-100 (Sigma), and 2% fish skin gelatin (Sigma) in PBS) for 30 min at RT. Samples were fluorescently stained for cell nuclei (DAPI (D1306, ThermoFisher Scientific); 10 min at RT) and actin filaments (Phalloidin (A12381, ThermoFisher Scientific); 1:200 in PBS; RT for 20 min). Samples were imaged using a multi-photon confocal microscope (Leica SP8; Germany).

## 3. Results

Irrespective of loading regime, none of the composites demonstrated signs of delamination between the PCL nanofibres and collagen gel layer over the seven-day period or upon manual handling, suggesting physical integration between these two phases.

**Figure 3:**
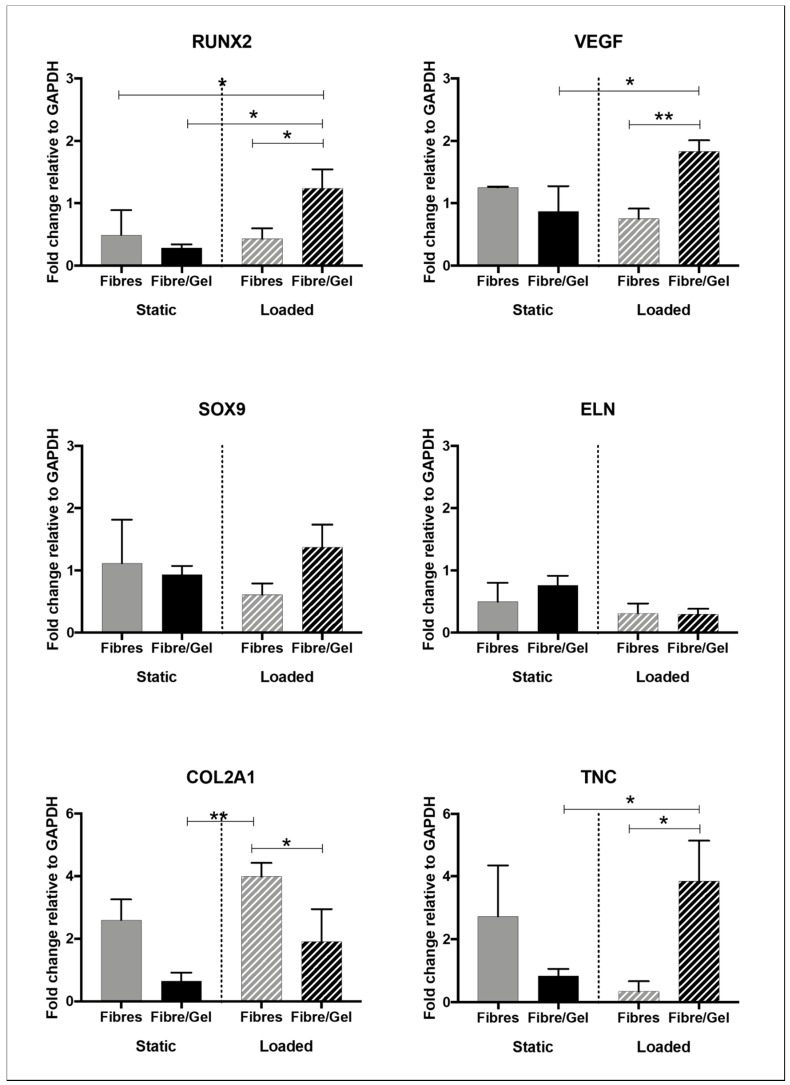
Gene expression for human mesenchymal stem cells demonstrating fold changes for specific genes relative to GAPDH after seven days for the four test groups. Data presented as mean ± standard error, two-way ANOVA, and post-hoc Tukey’s multiple comparisons test (* *p* < 0.05, ** *p* < 0.01).

qRT-PCR data demonstrated variable gene expression depending on the test group (Figure 3). Up-regulation in RUNX2, VEGF, and TNC was detected for mechanically loaded laminate composites when compared with loaded fibres. Similarly, mechanical stimulation had a positive effect on COL2A1 expression for fibre-only scaffolds. Composites and mechanical stimulation had no significant effect on SOX9 or ELN expression.

**Figure 4:**
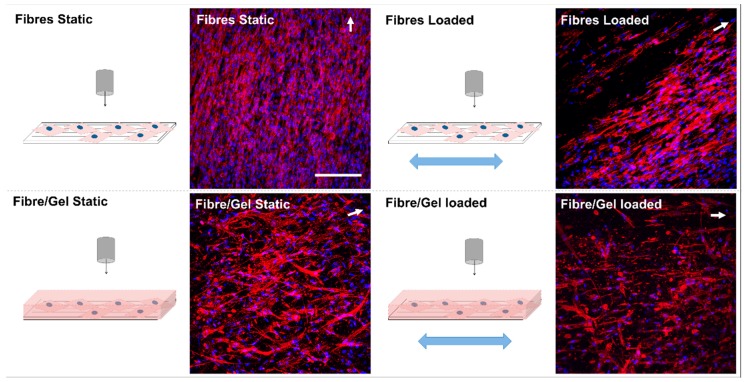
Confocal microscopy (z-stack birds-eye view) demonstrating human mesenchymal stem cell alignment relative to underlying fibre orientation (white arrows) for each group. Cells stained for nuclei (blue) and actin filaments (red). Scale bar = 200 μm.

After seven days, confocal z-stack images (Figure 4) demonstrated hMSCs were orientated parallel to the underlying fibre direction for both static and loaded fibre-only groups and cyclically stimulated fibre/gel composites. However, static culture of hMSCs within the fibre/gel laminate appeared less orientated, with actin filaments observed perpendicular to the main underlying fibre direction.

**Figure 5:**
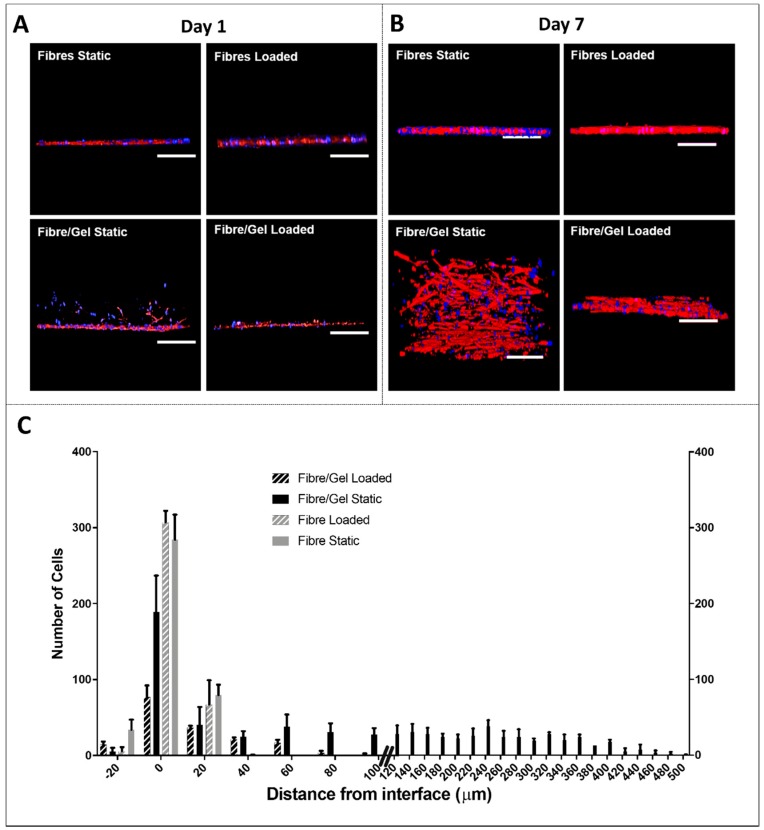
Transverse confocal microscopy images of human mesenchymal stem cells cultured on the four test groups after (**A**) one day and (**B**) seven days. Cells stained for nuclei (blue) and actin filaments (red). Scale bar = 200 μm. (**C**) Cell migration distance from fibre/gel interface after seven days.

The presence of a hydrogel layer affected cell movement (Figure 5). Transverse confocal images demonstrated hMSC migration from the fibre/gel interface to the upper gel phase for composites held under static conditions (at day 1), with greatest distance from this interface being 500 μm after seven days. In contrast, cells largely remained at the interface for cyclically stimulated composites. 

## 4. Discussion

Gene expression was analysed for several key tissue-specific markers to determine what effect the environment (fibres only or gel/fibre interface) and/or mechanical stimulation had on cell response (Figure 3). hMSCs cultured within the stimulated composite group exhibited an up-regulation for RUNX2, VEGF, and TNC with statistical significance when compared with static composites and stimulated fibres. This suggests hMSCs encapsulated on an aligned fibrous network and mechanically loaded (Group 4) directed towards several potential lineages: osteogenesis, angiogenesis, and tenogenesis. Interestingly, cartilage markers (SOX9 and COL2A1) demonstrated no benefit for hMSCs encapsulated within the 3D composite structure despite cartilage being a natural fibrous structure held within a gelatinous matrix [6]. However, we have previously demonstrated hMSC differentiation towards nucleus pulposus-like cells when encapsulated within UV-photocurable hydrogels and mechanically stimulated [5]. 

On the basis of similar findings with other cell types [7,8,9], hMSCs in contact with the fibre layer (with and without gel layer) preferentially orientated parallel to the main fibre axis, suggesting topographical cues were conferred to the cells (Figure 4). However, for unloaded composites, uniaxial cell orientation was lost for hMSCs that had migrated into the upper collagen layer because no structural organisation was present. Similarly, this migration demonstrates cell preference for a natural material environment over synthetic, which possesses no cell recognition sites for attachment [10,11,12] and instead weakly supports cell adhesion via adsorbed proteins [13]. hMSCs migrated up to 500 μm from the fibres’ surface to the collagen gel, suggesting cells were highly motile and sensed RGD-binding ligands [14], which could act as chemotaxant preferential molecules [15]. However, the majority of cells remained at the interface (Figure 5). Approximately 1% of cells were located 100 μm above the fibres within the collagen gel despite cyclical stimulation. This may be attributed to these cells experiencing loads greater than their cell-substrate adhesion strength, resulting in them breaking free from the fibre surface [16]. Despite this, hMSCs were predominantly located at the interface (75% of cells ± 20 µm from interface), suggesting cyclical stimulation was applied to both the gel and fibre phases as further indicated by the integration of the gel layer with the fibrous scaffold. This loading regime could have impacted the stiffness of the collagen gel and subsequently affected cell phenotype. Changes in matrix stiffness are known to impact cell phenotype, cytoskeleton, proliferation, and motility as well as drive stem cell differentiation towards specific lineages through subsequent changes in genetic and protein expression [17]. 

This paper demonstrates the competition or synergistic effect different stimuli can impose on hMSC phenotype. hMSCs prefer a natural, 3D environment over electrospun synthetic fibres alone, and they are particularly receptive to applied mechanical forces. This study emphasises the importance of developing biomaterial structures that are fit for purpose, i.e., consideration of the cellular environment (2D or 3D) and external stimuli, e.g., mechanical loading, needs to be incorporated (or dismissed) as appropriate for the structure’s function. This paper is the first to demonstrate a 3D, in vitro model, which could be fine tuned to better mimic the ECM of the tissue of interest and incorporate mechanical stimuli to direct stem cell behaviour. Further investigations would be required to evaluate the effectiveness of this bottom-up approach technology to produce neo-tissue that replicates the correct protein composition of the native tissue of interest. This model provides a basic platform to study cell phenotype in vitro and could be specifically tailored to further simulate the natural 3D tissue environment.
